# Plasmonic metafibers electro-optic modulators

**DOI:** 10.1038/s41377-023-01255-7

**Published:** 2023-08-22

**Authors:** Lei Zhang, Xinyu Sun, Hongyan Yu, Niping Deng, Feng Qiu, Jiyong Wang, Min Qiu

**Affiliations:** 1 College of Information Science and Electronic Engineering, Zhejiang University, Hangzhou, 310027 China; 2 Key Laboratory of 3D Micro/Nano Fabrication and Characterization of Zhejiang Province, School of Engineering, Westlake University, 18 Shilongshan Road, Hangzhou, 310024 Zhejiang China; 3 Institute of Advanced Technology, Westlake Institute for Advanced Study, 18 Shilongshan Road, Hangzhou, 310024 Zhejiang China; 4 College of Optical Science and Engineering, Zhejiang University, Hangzhou, 310027 China; 5 Hangzhou Institute for Advanced Study, University of Chinese Academy of Sciences, Hangzhou, 310024 China; 6 Ministry of Education Engineering Research Center of Smart Microsensors and Microsystems, School of Electronics and Information, Hangzhou Dianzi University, Hangzhou, 310018 China

**Keywords:** Metamaterials, Nanophotonics and plasmonics

## Abstract

Digitalizing optical signals through electric driving signals, electro-optic modulators (EOMs) are one of the cardinal elements in modern optical communications. Most of current EOM devices are targeting on-chip integrations, which routinely suffer from high coupling losses, complex optical alignments and single-band operations. In this study, we for the first time integrate a lumped EOM device on the endfaces of a single-mode optical fiber jumper for fast amplitude modulations. Profiting from ultrathin and high quality-factor plasmonic metasurfaces, nanofabrication-friendly and highly efficient EO polymers and coupling-free connections with fiber networks, our EOM is demonstrated to allow dual-band operations (telecom O band and S band) and high-speed modulations (~1 GHz at a bias voltage of ±9 V). This work offers an avenue to ‘plug-and-play’ implementations of EO devices and ultracompact “all-in-fibers” optical systems for communications, imaging, sensing and many others.

## Abstract (web summary)

Direct integration of an electro-optic modulator device on the endface of a single-mode fiber jumper for coupling-free treatments, dual-band operations (telecom O/S band) and high-speed modulations (~1 GHz).

## Graphical abstract

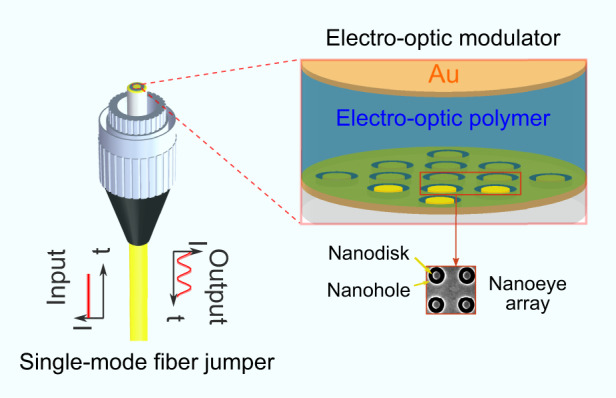

## Introduction

Electro-optic modulators (EOMs) are indispensable elements in the optical communication systems, which control the amplitude, phase and polarization of a light via external electric signals^1–3^. Aiming to realize ultracompact and high-performance EOMs, the most investigations nowadays target on-chip devices that combine semi-conductor technologies with state-of-art tunable materials^4–7^. The EO polymers, as one of the most outstanding candidates of tunable material have gained tremendous interests because of their large electro-optic coefficient r_33_, easy integrations and ultra-high bandwidths^8–12^. The r_33_ of EO polymer has been demonstrated to be over 100 pm/V^10,11,13^, which is almost three times larger than that of commonly used crystal-based system (e.g., LiNbO_3_)^14^, profiting from a strong Pockels effect supported by a large hyperpolarizability of chromophores induced by centrosymmetry breaking after an electric poling process^15^. The Pockels effect also allows a linearity of external electric field and a high energy efficient for an integrated EO device^16,17^. Combining the EO polymer with Mie resonator metasurfaces, a recent study demonstrated an on-chip EOM with the modulation frequency beyond gigahertz (GHz)^12^. Nevertheless, integrated EOMs, as an independent on-chip element, are commonly separated from light sources. Thus, extra interfaces couple the light from light sources to the waveguides of on-chip devices is indispensable. Two of widely used coupling schemes include edge coupling^1,2,18–20^ and grating coupling^21–23^, which suffer from limited integration densities and narrow-band operations, respectively. Besides, both coupling schemes require ultra-accurate alignments and complex encapsulations, making on-chip devices expensive for customers^24,25^.

To circumvent the coupling complexity and further reduce coupling losses, a feasible solution is to directly integrate EOMs within optical fibers, connecting EOM devices with light sources using standard interfaces of optical fibers. For the sake of all-fiber operations, hybrid configurations using tunable materials and specially possessed optical fibers, for instance, tapered optical fibers^25–28^, D-shaped optical fibers^24,29,30^ and photonic crystal fibers^31,32^, have been demonstrated to modulate the amplitude of guided light. However, tunable materials in most of such configurations are only allowed to interact with evanescent fields of the light. Thus, the effective thickness of tunable materials is restricted to the same order of penetration depth. This commonly leads to a poor modulation efficiency and a slow modulation speed. To our best knowledge, the fastest records of lumped and traveling-wave fiber-integrated EOMs are 250 MHz and 5 GHz for 3-dB bandwidths, respectively^29^.

**Fig. 1:**
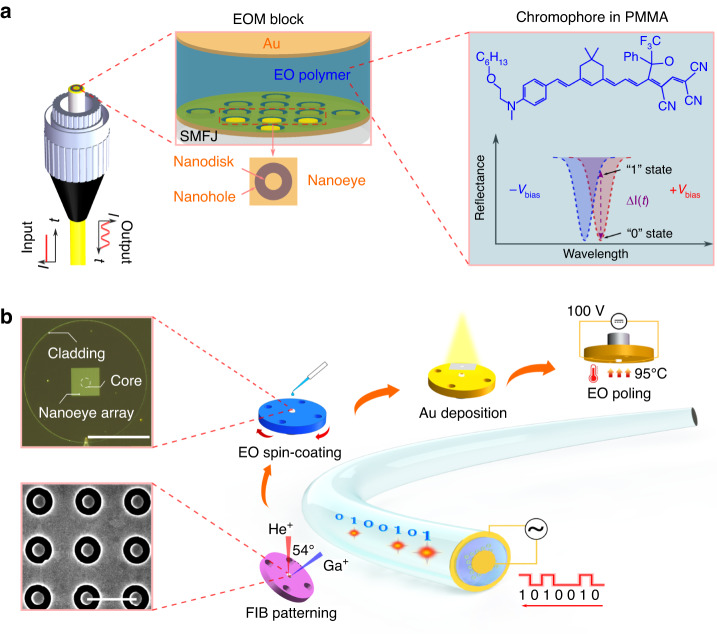
Schematics of configuration and nanofabrication of plasmonic metafibers for electro-optic modulators (EOMs). **a** Layer structure of plasmonic metafiber EOMs. The device consists of three layers: plasmonic nanoeye metasurfaces (bottom layer), EO polymer (middle layer) and a uniform Au film (top layer), which are integrated on the endface of a single mode fiber jumper (SMFJ). The inset shows the chemical structure of EO polymer and the working principle of EOMs. The reflectance amplitude of optical signals can be modulated between ‘open’ (logic ‘1’) and ‘close’ (logic ‘0’) states by applying bias voltages. **b** Nanofabrication flow to prepare the plasmonic metafiber EOMs. The top inset is an optical image of the metafiber after the spin-coating of EO polymer. The bottom inset is a scanning electron microscope (SEM) image of the plasmonic nanoeye array on the fiber enface. The scale bars in the optical image and SEM image are 62.5 μm and 850 nm, respectively

Alternatively, benefiting from state-of-art nanofabrication techniques, metafibers integrating well-defined metasurfaces on the optical fiber tips provide an unprecedented platform to manipulate the amplitude, phase and wavelength of the light^33–40^. The functionality of optical fibers has been expanded from conventional waveguides to ultracompact sensing^34,38^, ultrathin fiber lenses^37,39^, ultrafast fiber lasers^40^ and other lab-on-fiber technologies^35,36^ In this study, we demonstrate a first lumped EOM that is integrated on the enface of a commercial single-mode optical fiber jumper (SMFJ), as illustrated in Fig. 1a. The tunable material, that is EO polymer, interacts with the light in the longitudinal direction, leading to a μm scale of interaction length. The light-matter interactions are further enhanced by various resonant modes supported by rational designs of plasmonic metasurfaces on the fiber endfaces. Our EOM device is demonstrated to allow coupling-free treatment, multi-band operations and high-speed modulations.

## Results

Figure 1 shows the principal configuration and fabrication processes of the plasmonic metafiber EOM. As shown in Fig. 1a, plasmonic metafiber EOM consists of a bottom layer of Au plasmonic metasurface, a middle layer of EO polymer and a top layer of Au film. The unit cell of plasmonic metasurfaces is a nanoeye structure, which is a hybrid nanostructure of a nanohole and a nanodisc. The nanoeye metasurface serves as the bottom electrode^41^ in the EO modulation process, in addition to playing plasmonic roles during light-matter interactions. Besides, nanoeye features centrosymmetric so it is immune to the polarization rotation. The EO polymer we use is a mixture of chromophore and Poly-methyl methacrylate (PMMA), the chemical structure of which is shown in Fig. 1a (see “Synthesis of EO” from SI for details). Such an organic EO material provides a high compatibility with nanofabrication processes^42^. The effective refractive index of EO polymer can be tuned by applying bias voltages, which lead to a spectral shift of mode resonances. The amplitude of reflected light at designated wavelengths can thus be modulated with the same temporal response as external electric signals. The top layer serves as a nearly perfect mirror as well as the other electrode, forming a Fabry-Perot (FP) cavity together with the bottom Au metasurfaces. The whole plasmonic-organic hybrid EOM is integrated on the endface of a commercial SMFJ, as depicted in Fig. 1a.

Figure 1b shows the nanofabrication flow of plasmonic metafiber EOMs, which mainly includes nanopatterning of metasurfaces using focused ions beam milling (FIB), spin-coating of EO polymer, deposition of top Au electrode and electric poling of EO polymer. The detailed processes for FIB writing metasurfaces and spin-coating of organic films on the endfaces of commercial SMFJs were reported in another place^40^. Comparing with most of metafibers using bare fibers^33,34,37–39^, SMFJs provide a much larger operation area, making multilayer nanofabrication as well as electrode leadings at a single endface of a fiber feasible^43^. The bottom inset shows a scanning electron microscope (SEM) image of nanoeye structures at the fiber endface. The top inset shows its dark-field optical image after covering a layer of EO polymer. During the poling process, the device is heated above the glass-transition temperature (95 °C) of EO polymer and applied a DC bias voltage of 100 V. (see “Nanofabrication flow” from SI for details).

**Fig. 2:**
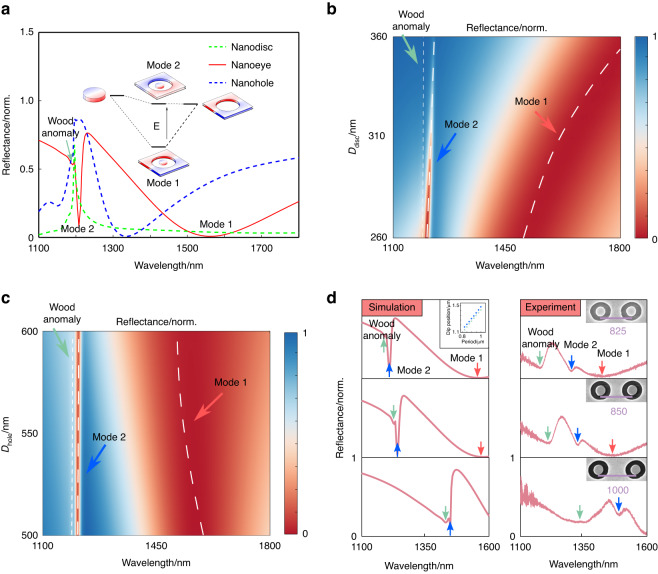
Plasmonic mode analyses of bare metasurfaces layer. **a** Theoretical reflection spectra and mode origins of nanoeye metasurfaces. The red solid line is the reflection spectrum of nanoeye arrays. Two modes appear at the spectrum: mode 1 and mode 2. The blue and green dashed lines are the reflection spectra of pure nanohole and nanodisc arrays, respectively. The diameters of nanohole and nanodisc are 550 nm and 280 nm, respectively. The period of the unit cell is 825 nm. The height of nanostructures is 55 nm. The inset shows the schematic energy diagram of hybrid modes 1 and 2. Dependences of the two hybrid modes in the reflection spectra of nanoeyes on the diameter of nanodisc (**b**) and nanohole (**c**). **d** Theoretical and experimental dependences of the two hybrid modes on the periodicity of nanoeye arrays. The inset shows periodicity dependences of mode 2 (blue circle) and Wood anomaly (green asterisk)

Figure 2 shows optical mode analyses of bare plasmonic metasurfaces. The unit cell is what we called Au nanoeye. We take one set of geometrical parameters for example to explain physical origins of plasmonic modes. The diameters of nanohole and nanodisc are 550 nm and 280 nm, respectively. The period of the unit cell is 825 nm. The height of nanoeyes is 55 nm. As shown in Fig. 2a, two dominant dips appear on the reflection spectra (red line) of nanoeye arrays, which are referred to as mode 1 and mode 2. The mode 1 and mode 2 are respectively the bonding and antibonding modes of the nanoeyes, coupled by the dipolar modes of nanodiscs (green dashed line) and nanoholes (blue dashed line)^44–46^. The inset in Fig. 2a shows a schematic energy diagram of such hybrid modes. The color contrast represents charge distributions of nanostructure arrays, which validates physical origins of mode 1 and mode 2 (see “Plasmonic hybridization model” from SI for details).

We further investigate dependences of the two modes on the geometrical parameters, so that the modes can be quantitatively tuned to expecting optical wavebands. Figure 2b, c shows dependences of the modes in the reflection spectra of nanoeyes on the diameter of nanodisc (b) and nanohole (c). It is observed that mode 1 is quite sensitive to the diameters, while mode 2 is barely affected. When the disc diameter increases from 260 nm to 360 nm, the dip of mode 1 shifts from 1512 nm to the wavelength outside the observation window. When the hole diameter increases from 500 nm to 600 nm, the dip of mode 1 exhibits a noticeable blueshift from 1572 nm to 1552 nm. We further investigate their periodic dependences. Figure 2d shows theoretical and experimental reflection spectra of nanoeyes as a function of period of the unit cell. It is seen that two modes redshift with the period. The mode 1 eventually shifts to the wavelength outside the interested optical window (1100–1600 nm) when the period is larger than 875 nm. The dip position of mode 2 changes from 1208 nm to 1240 nm and finally reaches 1448 nm in theoretical calculations, when the period is set 825 nm, 850 nm, and 1000 nm, respectively. Correspondingly, the experimental dip position of mode 2 redshifts from 1300 nm to 1329 nm and finally reaches 1490 nm.

We also notice that there is a small dip quite close to the mode 2, which is recognized to be Wood anomaly. The dip position of Wood anomaly and mode 2 are almost linearly proportional to the period we investigate, as shown in the inset. This anomaly is formed when the surface plasmons polaritons are excited by the incident light at the region of periodically metal nanostructures^47^. As can be seen from Fig. 2b–d, the Wood anomaly appears accompanying the mode 2 and follows a similar evolution tendency as the period of the unit cell to the mode 2. As can be seen from Fig. 2d, the experiment agrees well with the theoretical predictions except of a broader linewidth for the Wood anomaly.

**Fig. 3:**
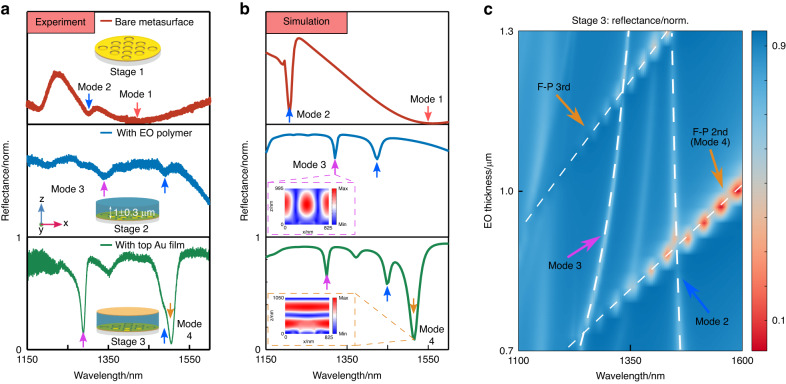
Optical modes analyses of the whole plasmonic metafiber EOM. Optical mode evolutions with sequential nanofabrication processes from experiments (**a**) and corresponding simulation (**b**). A new mode (mode 3) appears on the reflection spectra after the bare metasurface (top row, stage 1) is coated with a uniform layer of EO polymer (middle row, stage 2). Another new mode (mode 4) appears after covering the EO polymer with a uniform Au film (bottom row, stage 3). **c** Dependences of reflectance of mode 2, mode 3 and another two FP modes on the EO polymer thickness at the stage 3

Figure 3 shows the optical mode evolutions when the middle layer (stage 2) and the top layer (stage 3) are successively added to bare metasurfaces (stage 1). When the EO polymer is spin-coated over the plasmonic metasurfaces, the two coupled modes encounter large redshifts due to the increased dielectric constant of the surrounding media^48^. As shown in Fig. 3a, the dip of mode 2 shifts from 1300 nm to 1492 nm, while mode 1 redshifts totally outside our detection window. A new dip appears at 1338 nm, which is referred to as mode 3 hereafter. To figure out the physical origin of mode 3, the middle layer of EO polymer (thickness 940 nm) is conformally covered upon the nanoeye metasurface in the simulation model. The refractive index of EO polymer without bias voltage is measured by spectroscopic ellipsometry (WCMNF-2019-C004 brand, J.A. Woollam company) (see “Refractive indies of EO polymer” from SI for details). As first seen from the Fig. 3b, the mode 1 and mode 2 in the simulation agree with experimental results. We then check the optical electric field of mode 3, as shown in the inset from the middle row of Fig. 3b. The electric field of mode 3 is propagating along the transverse direction (x axis), denoting the mode 3 is a resonant waveguided mode due to the higher order (±1) of grating diffraction.

When the top Au film (55 nm) is further coated (stage 3), two features can be clearly observed from the reflection spectrum. First, the dip position of the two modes is slightly shifted: the mode 2 redshifts, while the mode 3 blueshifts. Second, the extinction ratios of the mode 2 and mode 3 are significantly augmented by 9 and 7 times respectively, promoting a higher sensitivity to the bias voltage for amplitude-modulating EOMs. The bottom row of Fig. 3b shows the corresponding simulation. A new dip appears at the longer wavelength aside mode 2, which is referred as to mode 4 hereafter. The dip at 1505 nm observed from the experiment is in fact a coupled mode between the mode 2 and mode 4. Hereafter, we refer this mode to as coupled mode for convenience. This could explain the slight shoulder appears at 1500 nm from the experiment, shown in the bottom row of Fig. 3a. To understand the physical origin of mode 4, we further calculate the electric field distribution. As shown in the inset from the bottom row of Fig. 3b, interfered patterns are propagating in the longitudinal direction and sandwiched between the top and the bottom layers. We observe no confined fields from the bottom metasurfaces. Thus, the mode 4 is predominantly determined by the cavity length, that is the thickness of EO polymer. To further confirm the origin of mode 4 and achieve a precise manipulation of the positions of modes 2, 3 and 4, cavity length dependences are systematically studied. As shown in Fig. 3c, as the thickness of EO polymer increases, the mode 2 almost remains the same position, while the mode 3 slightly redshifts in a nonlinear manner. In addition to the mode 2 and mode 3, there are two discrete modes, which have linear dependences on the cavity length. These two modes origin from the interferences of electric field in the cavity, which are thus referred as to FP modes. Indeed, FP modes follow a function of Φ_metasurface_ + Φ_Au mirror_ − 4πH_EO_/λ = 2Nπ, where H_EO_ is the thickness of EO polymer, N is the integer, and Φ_metasurface_ and Φ_Au mirror_ denote the phase accumulations introduced from the metasurface layer and the top Au film, respectively^49^. Thus, the upper and the lower linear modes are identified to be the 3rd and 2nd orders of FP mode, respectively. The mode 4 is in fact the 2nd order of FP mode of such a metafiber EOM.

**Fig. 4:**
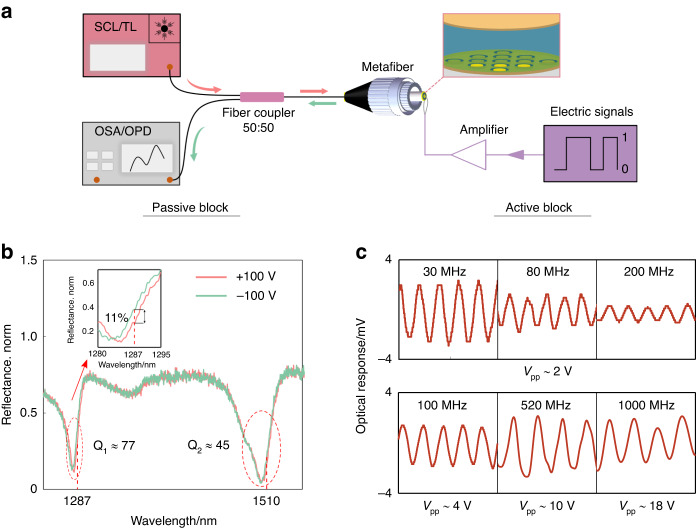
Modulation performances of the plasmonic metafiber EOM. **a** Experimental setups for direct current (DC) and alternative current (AC) modulation characterizations, which consist of a light source, a metafiber EOM, a signal generator with a voltage amplifier and an optical detector. Depending on DC or AC modulation tests, the light source could be selected as either a supercontinuum laser (SCL) or a tunable telecoms laser (TL), the signal generator uses either static bias voltages or radio frequency (RF) bit signals, and the optical detector is either an optical spectrum analyzer (OSA) or a fast oscilloscope with photodetector (OPD). **b** DC modulation performance of the plasmonic metafiber EOM. The reflection spectrum of plasmonic metafiber undergoes a redshift when the DC bias voltage changes from −100 V (green line) to +100 V (red line). The inset shows a modulation depth of 11% at the wavelength of 1287 nm. **c** AC modulation performances of plasmonic metafiber EOM at different driving frequencies. Top row shows the temporal responses of an oscilloscope under a constant bias voltage of ±1 V; the bottom row shows the temporal responses of an oscilloscope with nearly the same amplitude of output signals

Following passively optical characterizations, EO modulation tests are conducted using our plasmonic metafiber EOMs. Figure 4 shows experimental setups and measurements of modulation performances. Figure 4a shows the experimental setups to characterize the direct current (DC) and alternative current (AC) performances, which mainly consist of a light source, a metafiber EOM, a signal generator with a voltage amplifier and an optical detector. The light source could be chosen as either a supercontinuum laser (operating wavelengths: 400–2400 nm) or a tunable laser (operating wavelengths: 1260–1360 nm and 1500–1600 nm). A 50:50 fiber coupler is used to connect the metafiber EOM with the light source and the optical detector. Two electrodes connecting the bottom metasurface layer and the top Au film layer are extended using Ag wires (diameter: 60 μm). The signal generator (SMT03, ROHDE&SCHWRZ, maximal signal frequency: 3 GHz) with a tunable V_pp_ (−140–16 dBm) is used to supply radio frequency (RF) signals. Depending on the bias voltage, an optional RF amplifier (PA-VLUHF-43, 100 kHz ~ 1 GHz) with a constant gain of 40 dB is employed to amplify the initial electric driving signals. During the EO modulation tests, the driving signals are transmitted through the two Ag wires. The optical detector uses either an optical spectrum analyzer or a fast oscilloscope with a photodetector. For the DC characterizations, a super continuum laser is chosen as the light source and an optical spectrum analyzer is used to detect output optical signals. Figure 4b shows reflection spectra of the metafiber EOM in the applications of DC voltages of +100 V (red line) and −100 V (green line). Two sharp dips locating at O band (1283 nm) and S band (1500 nm) can be clearly observed, the quality factors (Q factors) of which are calculated as 77 and 45, respectively. The two dips are regarded to be the mode 3 and the coupled mode, as analyzed in Fig. 3. When the DC voltage changes from −100 V to 100 V, the dips shift ~3 nm toward longer wavelengths. The deduced in-device r_33_ is 15 pm/V, and the refractive index change at the operation wavelengths 1287 nm and 1510 nm is 0.0041 and 0.0038, respectively (see “Refractive indies of EO polymer” from SI for details). A parameter S = Δλ/(ΔFWHM*Δn)^2^ is introduced here to quantify resonance sensitivity to the bias voltage by taking both the spectra shift and the bandwidth into account,where S is normalized sensitivity, Δλ is the wavelength change of the resonance dip, ΔFWHM is the full-width at half-maximum of the resonance and Δn is the refractive index change at the resonance dips (for 1283 nm Δn is 0.004, and for 1500 nm Δn is 0.0038). Therefore, the normalized sensitivity for the two resonances locating at 1283 nm and 1500 nm is 44 RIU^−1^ and 23.6 RIU^−1^, respectively, which are slightly larger than the value (17 RIU^−1^) of plasmonic EOM reported^2^. The modulation depths are ~11% at the telecom laser wavelengths of 1287 nm and 1510 nm, as shown in the inset of Fig. 4b.

For the AC characterizations, a tunable laser working at telecom wavebands is chosen as the light source and the optical spectrum analyzer is replaced with a fast oscilloscope with a photodetector. Sinusoidal waves are generated to be driving signals. Thermal effects of EO polymer induced by the optical absorptions and electric powers can be neglected. (see “Thermal effects analysis” from SI for details) A series of modulation tests are performed to verify the high-speed operation of metafiber EOM. As can be seen in the top row of Fig. 4c, under a constant bias voltage (V_pp_ 2 V), the highest modulation frequency is demonstrated up to 200 MHz at both O band and S band. The amplitude of output signals decreases with the modulation frequency due to the reflection loss of signal power. The impedance mismatch of RF probe and electric cables become more obvious at higher RF frequencies. In other words, the actual power the metafiber EOM receives is much smaller than the input nominal power of signal generator^50^. In case to achieve a constant signal-noise ratio of receiving signals, an increasing bias voltage is required as the modulation frequency to compensate the reflection loss. In such cases, an RF amplifier is applied to amplify the power of input signals. As can be seen from the bottom row of Fig. 4c, the V_pp_ is required to increase from ~4 V to ~18 V if the modulation frequency increases from 100 MHz to 1000 MHz.

## Discussion

**Fig. 5:**
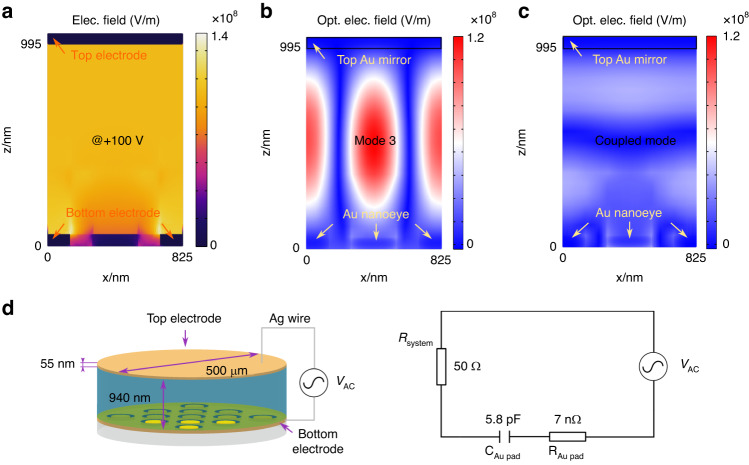
Theoretical evaluations of modulation speed of plasmonic metafiber EOMs. **a** Electric field distribution of metafiber EOM under a DC bias of +100 V. **b**, **c** Optical electric field distributions of metafiber EOM with resonant wavelengths of mode 3 and the coupled mode. **d** Geometrical parameters of metafiber EOM and the equivalent RC circuit

We then compare the overlapped field between the biased electric field and optical field in the EO polymer layer to assess which mode could support better modulation performances. As seen from Fig. 5a, under a DC bias voltage of +100 V, the top and bottom layer form an equivalent capacitor with a nearly uniform electric field inside. Even though the nanodiscs are insulated from the bottom electrode, gaps around the nanodiscs are still filled with intense electric fields, which enables the modulation of EO polymer around the nanodiscs. By applying a DC bias voltage of +100 V, the optical electric fields of mode 3 and the coupled mode almost retain their respective features as the cases without applying the bias voltage, as shown in Fig. 5b, c. However, the dip position of interested modes undergoes a redshift. The mode 3 redshifts from 1300 nm to 1302.5 nm, and the coupled mode between the mode 2 and mode 4 redshifts from 1487.5 nm to 1490 nm. Although the two modes have the same amount of dip shift, the extinction ratio change of mode 3 (Q ≈ 144) is larger than that of the coupled mode (Q ≈ 25), indicating a better signal-noise-ratio if the same driving voltage is applied. This can be further confirmed by comparing the overall electric field intensity in the EO polymer layer. The averaged electric field intensity of the mode 3 in a unit cell is 1.6 times stronger than that of the coupled mode, denoting that EO polymer could be more effectively excited to obtain a larger dip/peak position shift at high bias voltages. From the aspect of nanofabrication, the mode 3 is also preferable. Being a resonant waveguide mode, the mode 3 predominantly depends on the period of the metasurfaces, which can be precisely controlled by using current planar technologies. The coupled mode locating around 1490 nm, however, strongly depends on the thickness of EO polymer. The spin-coating of EO polymer on the fiber endface with high precision and uniformity is always a challenge. Thus, the mode 3 is a better option for practical use in comparison with the coupled mode if the repeatability of single-band EOM device is a concern.

As the first demonstration of a lamped EOM integrated onto the fiber tip, the modulation efficiency can be further improved and bias voltage can be reduced from the following aspects: First, optical modes with higher Q factors, for instance bound states in the continuum^51^ and Mie resonances^12^, could be introduced to our device system by replacing the plasmonic metasurfaces with dielectric metasurfaces. Studies have shown that the Q factor of such optical modes can reach as high as 1950^51^, which is more than one order higher than that of current case. The spectral bandwidth could be largely reduced, so that the sensitivity of EOM devices can be significantly augmented in DC modulations. Second, the EO coefficient of current EOM device is evaluated to be around 15 pm/V, which is much lower than the values of the best performances of EO polymers^10,13^. A higher EO coefficient could be reached if the first order hyperpolarizability is enhanced by, for example, enlarging the conjugation length or modifying appropriate electron donor or acceptor on the groups of chromophores^15^. It is also worth noting that it is always a trade-off between the choices of high Pockels coefficient and high stability. Sensitive EO polymer undergoes easier degradations in the absence of poling field to randomize the chromophores to reach the maximum entropy steady state^52^. Efficient solutions to alleviate such degradations are establishing stabler links between the guest chromophores and host chains. For instance, hydroxyl groups could be introduced into the host polymer or the chromophores to form hydrogen bond among the polymetric network^53^ or between the host and guest^54^, restricting the mobility of chromophore and thus improving the long-term stability. The host-guest system could be replaced by the side-main, main-chain and the cross-link systems if stability is the key concern^53^.

Furthermore, for the lumped EOM device, we also evaluate the theoretical 3-dB bandwidth f_3dB_ of the metafiber EOM. f_3dB_ can be calculated using the equation: (1/f_3dB_)^2^ = (2πτ)^2^ + (2πRC)^2^, where τ is the photon lifetime, R and C are the resistance and capacitance of the device system, respectively^55^. The plasmonic-organic hybrid structure can be regarded as a parallel-plate capacitor, as depicted in the left of Fig. 5d. The capacitance of metafiber EOM is calculated as C ≈ 5.8 pF. The resistance of system (R_system_) and Au pads (R_pad_) is evaluated as 50 Ω and 7 nΩ, respectively (see “Equivalent circuits calculation” from SI for details). Therefore, theoretical f_3dB_ is estimated about 550 MHz, overweighting the best performance of current lumped fiber-integrated EOMs. To achieve such a high f_3dB_, as seen from Fig. 4c, an approximate bias voltage of ± 5 V is required to apply to the source signals. To further promote the modulation performances, a proper impedance matching for the EOM device is required. Considering a considerable mismatch of impedance between the source and the load in current system, extra resistances need to be introduced to the modulator side to match the 50 Ω electrical network. This might be realized by a proper electrical design. For instance, using the coplanar electrode configuration^12^ replaces the current parallel electrode configuration or a segmented transmission line^56^ is introduced. A much higher electrical bandwidth and modulation efficiency can be achieved by using traveling-wave electrode design, instead of lumped electrode scheme, in which there is no limitation on RC time constant^57^. This could be research topic of our future work.

In summary, we for the first time integrate a lumped EOM device on the endfaces of a single-mode optical fiber jumper for fast amplitude modulations. The spectral amplitude and quality-factor of passed light are well controlled using a well-defined plasmonic-organic hybrid configuration. By rational designs of various plasmonic modes, resonant waveguided modes and FP modes, tunable dual-band operations can be achieved in telecom O band and S band. The modulation speed of metafiber EOM can reach as high as 1000 MHz with a bias voltage of ±9 V, which is comparable to the best performance for lumped fiber-integrated EOMs. All in all, our device is expected to further reduce the coupling losses in fiber networks of optical communications and to realize “all-in-fibers” operations for other optical systems.

## Materials and methods

All details about the EO polymers, nanofabrication, plasmonic hybridization simulation and equivalent circuits calculation are provided in the Supplementary information.

### Supplementary information

Supplementary Information for Plasmonic Metafibers Electro-optic Modulators
