# Supplementary material for: Plasmonic metafibers electro-optic modulators
Source: Light Sci Appl. 2023 Aug 22;12:198. doi: 10.1038/s41377-023-01255-7 (PMC10444839; doi:10.1038/s41377-023-01255-7)
Supplement: Supplementary file 1 — Supplementary Information for Plasmonic Metafibers Electro-optic Modulators [file 41377_2023_1255_MOESM1_ESM.docx]

**Supplementary Information for**

**Plasmonic Metafibers Electro-optic Modulators**

**Lei Zhang^1,3,4,#^, Xinyu Sun^2,3,4,#^, Hongyan Yu^2,3,5^, Niping Deng^3,4^, FengQiu^5^, Jiyong Wang^6*^, Min Qiu^3,4*^**

**# Equal contributions**

***Correspondence to: jiyongwang@hdu.edu.cn;**

[qiumin@westlake.edu.cn](mailto:qiumin@westlake.edu.cn)

1. **Synthesis of EO polymer**

**Figure S1. Synthetic route of chromophore.**

The guest-host chromophore that has the Pockels effect was synthesized following the recipe shown in Figure S1. In the initial step, donor compound 3 was synthesized by using compound 1 and compound 2. Next, compound 4 was synthesized through Knoevenagel condensation of compound 3 and isophorone. Subsequently, compound 5 was obtained through the Wittig-Horner reaction between compound 4 and diethyl(cyanomethyl)phosphonate. Following this, compound 6 with aldehyde group was synthesized by DIBAL-H-involved reduction of the nitrile group of Compound 5. Finally, the chromophore in the form of green solid particle was obtained by condensation between compound 6 and the acceptor (CF_3_-TCF). We then dissolved the chromophore and PMMA particles in the [cyclopentanone](http://dict.youdao.com/w/cyclopentanone/#keyfrom=E2Ctranslation) in the mass percent of 5:10:75, which is optimized for the formation of a uniform EO layer on the fiber tip, seen from the top inset of Figure 1(b) in the main text.

1. **Nanofabrication flow**


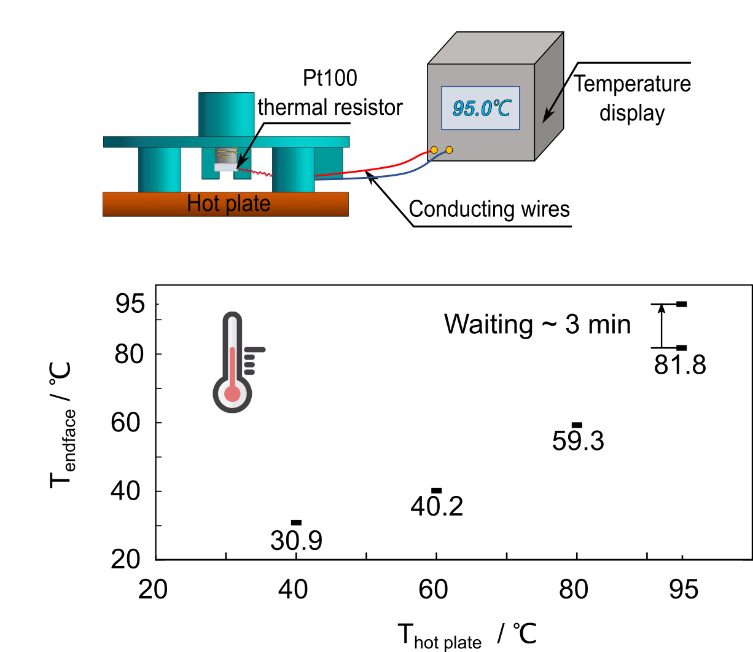


**Figure S2. Setups for temperature calibration of the optical fiber endface.**

All reported devices were processed on the single mode optical fiber jumpers (SMFJ), which could be easily connected with the custom-built translating chamber (for FIB patterning) and rotating chamber (for spin-coating and soft backing). More details could be found in our previous work (*1*). After spin-coating of EO, the metafibers were put into a vacuum drying chamber for 8 hours to further volatile the solvent. The Au deposition was performed using a mask to avoid the short-circuits between the top and bottom electrode during the physical vapor deposition process. A hole (diameter: 500 μm) is premanufactured through the laser direct writing, and the hole is concentrically aligned with the core region with the help of optical microscopy, as shown in Figure 1b. During EO poling process, an adaptor with four pillars was used to stably hold the fiber for a constant heating. There was a slight temperature difference between fiber-tip surface and the hot plate. A Pt100 platinum thermistor was used to calibrate the actual temperature of the fiber tip, as shown in Figure S2. At least 3 min of hating time was needed if the fiber tip reached the same level of the hot plate (tested in 95 ℃). To apply the DC bias voltage, two Ag wires (diameter: 60 μm) were respectively connected the top and bottom electrodes by using the conductive silver paste. The metafiber was first heated above glass-transition temperature (95 ℃) of EO polymer. Following this step, the poling voltage was applied from 0 V to 100 V continuously in the step of 1 V s^-1^, and the sample was subsequently cooled down to the room temperature with keeping the maximum poling voltage (*2*). The choice of poling voltage 100 V is mainly determined by empirical tests. When the poling voltage is below 80 V, the Pockels effect is largely limited (~20 % of the maximum value of Pockels coefficient) due to the thermal agitations (*3*). When the poling voltage increases from 80V to 100 V, the Pockels coefficient can reach as high as 80 % of the maximum value. If the poling voltage continues increasing, the Pockels coefficient increases in a much slower rate, in comparison with the poling voltage between 80 V and 100 V. We also test that the dielectric breakdown might occur if the poling voltage is increased to ~110 V, corresponding to a field strength of 117 V μm^-1^. Thus, the poling voltage of 100V is a trad-off between a larger Pockels effect and a lower risk of dielectric breakdown in our system (*4*).

1. **Plasmonic hybridization model**


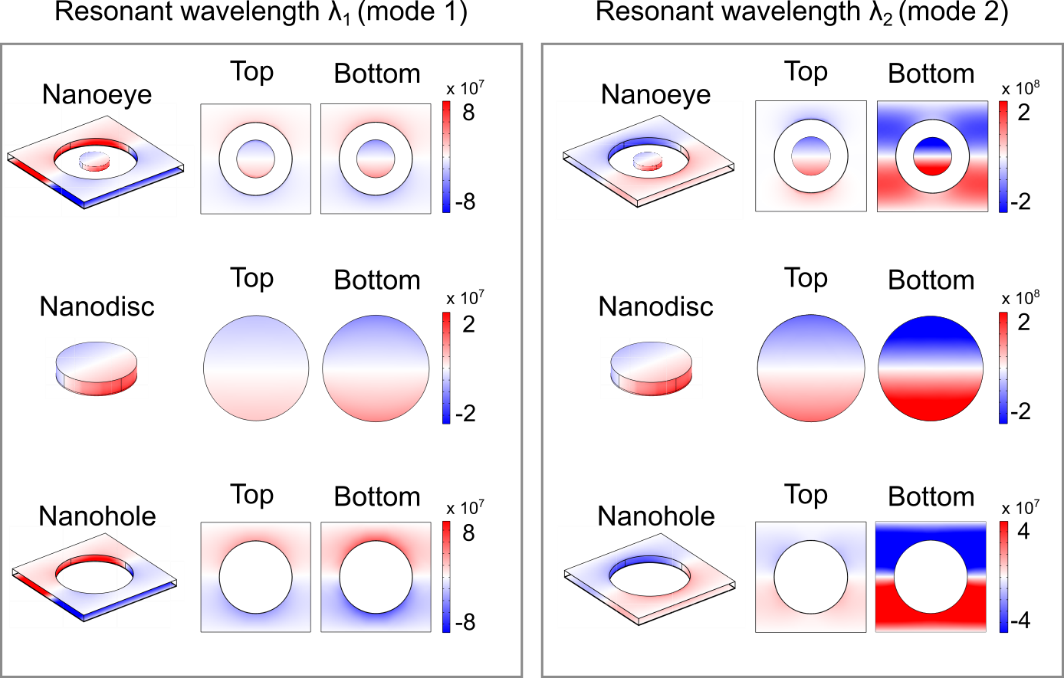


**Figure S3. Calculation of in-plane surface charge distributions of nanoeye, nanodisc and nanohole at resonant wavelengths *λ*_1_ and *λ*_2_, respectively.**

To fully understand the origin of mode 1 and mode 2, we calculated the in-plane surface charge distributions at both top and bottom surfaces of nanoeye, nanodisc and nanohole arrays at the resonance wavelengths (*λ*_1_ for mode 1 and *λ*_2_ for mode 2). As seen from Figure S3, at resonant *λ*_1_, the charge distributions at top and bottom surfaces of nanodisc and nanohole have the same orientations. When they are concentrically placed together, a typical super-radiant mode is formed, featuring a wide resonance dip in the reflectance spectrum, as depicted in the inset of Figure 2(a). On the other hand, at resonant *λ*_2_, the charge distributions have opposite orientations and thus an antiparallel dipolar interaction reduces the total dipole momentum and suppresses the radiative loss, resulting in a typical sub-radiant character.

1. **Refractive indies of EO polymer**

**
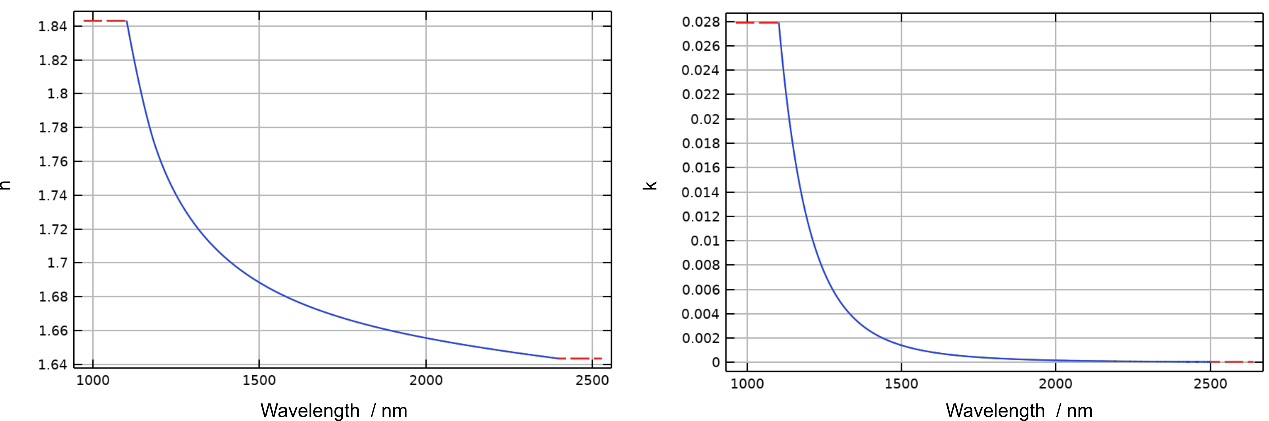
**

**Figure S4 The refractive indices of EO polymer from 1100 nm to 2400 nm measured by spectroscopic ellipsometry.**

Dispersive refractive index of EO polymer is measured by a spectroscopic ellipsometry (WCMNF-2019-C004 brand, J.A. Woollam company), the real part and the imaginary part of which are shown in Figure S4. As shown in the inset of Figure 4(b), the deduced in-device *r*_33_ is 15 pm V^-1^ if a bias voltage of 100 V is applied. We can use the formula Δ*n* = 0.5**r*_33_**n*_0_^3^**E* to evaluate the change of refractive index, where *n*_0_ is the refractive index in the absence of bias voltage and *E* is the electric field within the EO polymer layer along the longitudinal direction (*5*). The refractive index *n*_0_ at the operation wavelengths 1287 nm and 1510 nm is 1.7278 and 1.6871, respectively. *E* equals to 106 V μm^-1^ if a bias voltage of 100 V is applied to the electrodes. The change of refractive index Δ*n* can be evaluated to be 0.0041 and 0.0038 for the operation wavelengths of 1287 nm and 1510 nm, respectively. Similarly, Δ*n* at the dip position of 1283 nm and 1500 nm can be evaluated to be 0.004 and 0.0038, respectively.

1. **Thermal effects analysis**

We first consider the thermal effect induced by the optical absorption. To quantify the temperature change induced by the laser, we build a Multiphysics mode in COMSOL to couple the optical field and the thermal field in our modelling system. The thermal parameters of the materials are listed in Table 1 *(6, 7*). The geometric model of a unit cell is shown in Figure S5(a).


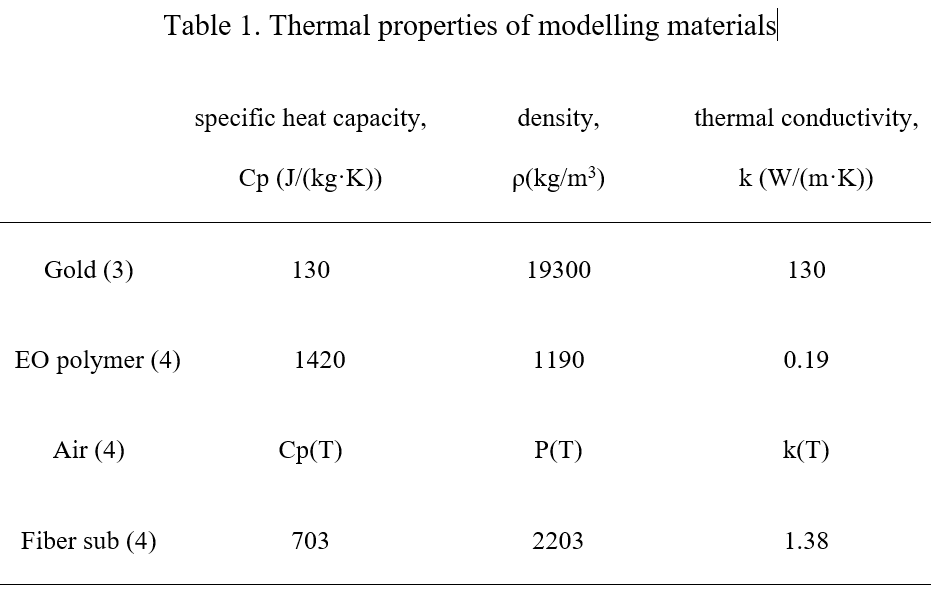


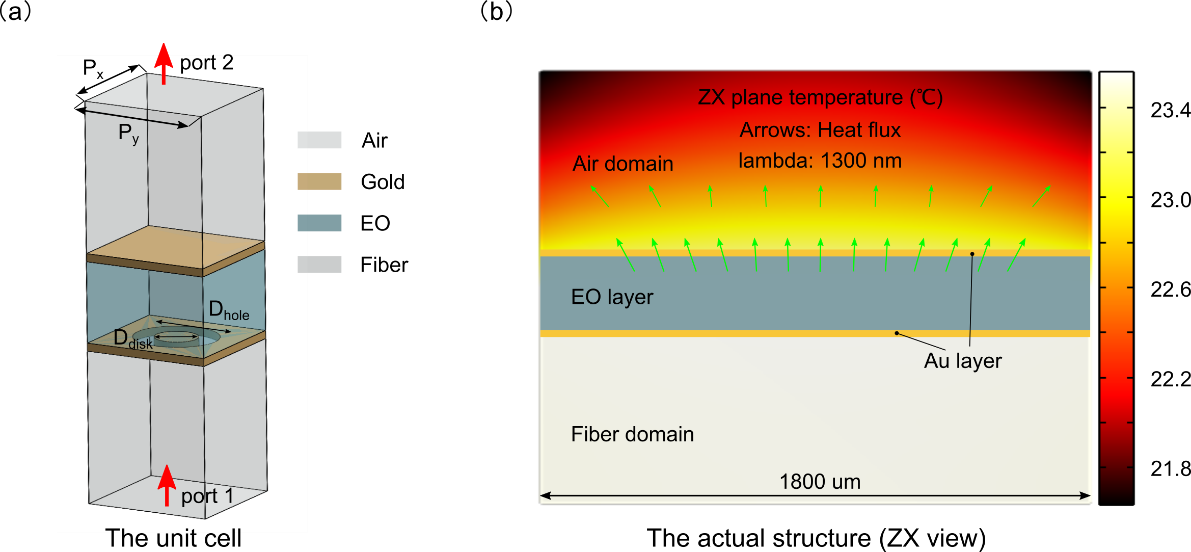


**Figure S5 (a) Geometric model in COMSOL. (b) Equilibrium temperature distribution in the cross-section (XZ plane) of the modelling system. The arrows indicate the heat flux.**

As shown in Figure S5(a), a plane wave with the power *P*_in_ = 8.3 μW is incident from the port 1, the power of which corresponds to the 1.58 mW CW laser illustrating on the core region (9 μm) of a single-mode fiber in our experiment. Periodic conditions are set at the boundaries. The background temperature is set 20 ℃. A heat flux boundary is set at the top boundary in the thermal field (module of heat transfer in solids) to represent the convective heat exchange between the EO polymer and air, where the heat transfer coefficient h equals 10 W m^-2^ K^-1^ (*8*). We then numerically find out how much of energy is absorbed at each resonance wavelength in the EO polymer layer of a unit cell by making a volume integration of the electromagnetic wave heating (*h*t.*Q*_tot_). Considering the convective and irradiative heat exchange occurs at the whole structure instead of a unit cell, we define the power transform coefficient *P*_c_ to describe the ratio of heat power generated within EO polymer over the initial input light power. Thus, *P*_c_ = *h*t.*Q*_tot_ / *P*_in_. For the mode 3, *P*_c_ = 0.225, while for the coupled mode, *P*_c_ = 0.024. An actual configuration of our EOM is then built in another COMSOL model for only solving the thermal field. The configuration is simplified into a EO layer sandwiched by the top and bottom Au layer with the diameter of 1800 μm so that the actual convective and irradiative heat exchange could be guaranteed. Again, the heat flux boundaries are set at the adjacent boundaries of the air domain to represent the convective heat exchange. The heat source is set within the domain of EO polymer, where *Q*_tot_ = 1.58 mW · *P*_c_ / (*π* · (1800 μm)^2^ · 940 nm). Figure S5(b) shows equilibrium temperature distribution of *XZ* plane at resonance wavelength of 1300 nm. The maximum temperature increase for mode 3 is about 4 ℃, while for the coupled mode such an increase is less than 1 ℃.

We next consider the thermal effect induced by the electric power. Similarly, we build a Multiphysics model in COMSOL to couple the electric field and thermal field in our modelling system. In such a Multiphysics model, the top electrode is set as an equipotential body and the bottom electrode is set as the ground, as shown in Figure S6(a). No periodic condition is added, so the temperature increase in the unit cell can be regarded as that in the actual configuration. Like the optothermal case discussed above, a heat flux boundary is set at the top boundary to represent the convective heat exchange. As shown in the Figure S6(b), the maximum temperature increase within EO polymer is less than 0.2 ℃ when the *V*_in_ is set as 9 V, which is the case of our experiment in the main text.


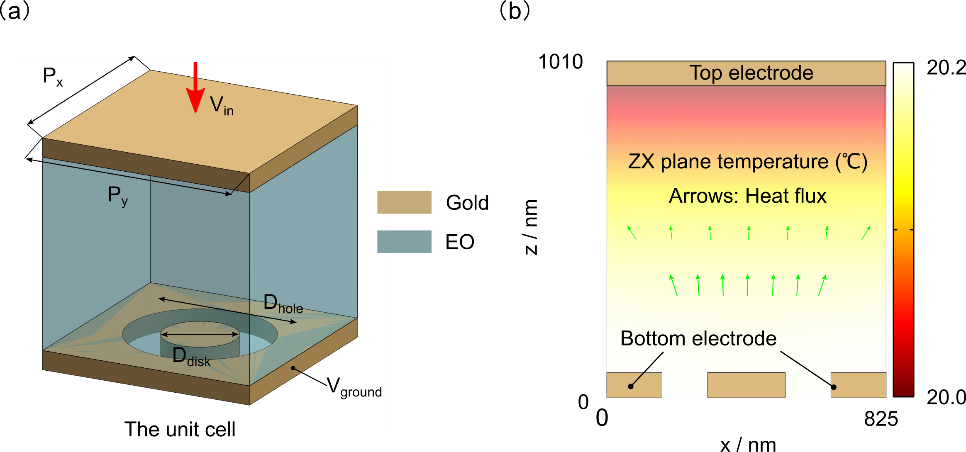


**Figure S6 (a)** **Geometric model in COMSOL. (b) Equilibrium temperature distribution in the cross-section (*XZ* plane) of the modelling system. The arrows indicate the heat flux.**

In summary, if the thermal effects induced by the optical absorption and electric power are both considered, the temperature in the thermal sensitive layer of EO polymer increases less than 5 ℃, which has negligible impact on the electrooptic modulator performances.

1. **Equivalent circuits calculation**

The hybrid structure is served as a parallel plate capacitor as depicted in Figure 5(b). The capacitance is calculated in according to *C = ε*_0_*ε*_r_*A/d*, where *ε*_0_ is the permittivity of vacuum, *ε*_r_ is the relative permittivity of EO polymer, *A* is the area of plate overlap, and *d* is the distance between two plates. Here, *ε*_r_ is 3, *A* is determined by the top electrode whose diameter is 500 μm, and *d* is 940 nm, which results in *C* ≈ 5.8 pF. For evaluation of the resistance, 50 Ω for the whole system is taken into account. As for the Au electrode, we use the formulae *R*_pad_ *= ρ*_gold_*L/S*, ρ_gold_= 2.44·10^-8^ Ω·m, *L* is the thickness of Au film (55 nm), and *S* is the area of Au film (*D*_top_ = 500 μm, and *D*_bottom_ = 1800 μm), so the resistance of Au pad is calculated as about 7 nΩ. To calculate the 3 dB and 6 dB bandwidths of the metafiber EOMs, we derive the formula from the RC circuit

$\begin{aligned} \sqrt{\frac{P_{\mathrm{out}}}{P_{\mathrm{in}}}}= \frac{1}{\sqrt{1+\left( \frac{2\pi f}{2\pi f_{c}} \right)^{2}}}\#\left( 1 \right) \end{aligned}$

where *P*_out_ is the output power, *P*_in_ is the input power, *f* is the driving frequency of the signal generator and *f*_c_ is the cut-off frequency of the device under test. When the *P*_out_ is decreased to half of the *P*_in_, the *f* is equal to *f*_c_, and we get the 3 dB cut-off frequency. When the *P*_out_ becomes a quarter of the *P*_in_, we get the 6 dB frequency, corresponding to a relation of *f*_6dB_ = $\surd3$*f*_c_ = $\surd3$*f_3_*_dB_.

Reference

1. L. Zhang *et al.*, ‘Plug-and-play’ plasmonic metafibers for ultrafast fibre lasers. *Light: Advanced Manufacturing* **3**, 45 (2022).
2. X. Sun *et al.*, Electro-optic polymer and silicon nitride hybrid spatial light modulators based on a metasurface. *Optics Express* **29**, 25543-25551 (2021).
3. J. W. Wu, Birefringent and electro-optic effects in poled polymer films: steady-state and transient properties. *JOSA B* 8, 1 (1991).
4. J. W. Wu, Birefringent and electro-optic effects in poled polymer films: steady-state and transient properties. Journal of the Optical Society of America B, 8, 1 (1991).
5. Weigand H., et al. Enhanced Electro-Optic Modulation in Resonant Metasurfaces of Lithium Niobate. *ACS Photonics* **8**, 3004-3009 (2021)
6. X. Chen, Y. Chen, M. Yan, M. Qiu, Nanosecond photothermal effects in plasmonic nanostructures. *ACS Nano* **6**, 2550-2557 (2012).
7. COMSOL Multiphysics, 5.6 version, built-in materials library.
8. Y. Zhang et al., Electrically reconfigurable non-volatile metasurface using low-loss optical phase-change material. *Nature Nanotechnology* **16**, 661-666 (2021).
